# Supplementary material for: Higher pNRF2, SOCS3, IRF3, and RIG1 Tissue Protein Expression in NASH Patients versus NAFL Patients: pNRF2 Expression Is Concomitantly Associated with Elevated Fasting Glucose Levels
Source: J Pers Med. 2023 Jul 18;13(7):1152. doi: 10.3390/jpm13071152 (PMC10381647; doi:10.3390/jpm13071152)
Supplement: Supplementary file 1 [file jpm-13-01152-s001.zip › jpm-2378563-supplementary.pdf]

Supplementary Table S1

Immunohistochemistry antibodies and staining protocols

Antibody	Company	Clonality	HIER	Dilution	Incubation	Detection	Company	Incubation	DAB	Hematoxylin
Vimentin	Dako	Ms. mono.	pH 9.0; 20 min. WB 96°C	/500	60min. RT	ZytoChem Plus AP	Zytomed	Kit	2x5min.	5min.
Active Caspase 3	Cell Signaling	Rb. poly	pH 9.0; 20 min. WB 96°C	/50	60min. RT	ZytoChem Plus AP	Zytomed	Kit	2x5min.	5min.
Ki67	Roche Ventana	Rb. mono.	pH 9.0; 20 min. WB 96°C	undiluted	60min. RT	ZytoChem Plus AP	Zytomed	Kit	2x5min.	5min.
IRF3	Invitrogen	Rb. poly	pH 9.0; 20 min. WB 96°C	/50	30min. RT	Polyview Plus AP Rb	Enzo		2x5min.	5min.
RIG1	Invitrogen	Rb. poly	pH 9.0; 20 min. WB 96°C	/200	30min. RT	Polyview Plus AP Rb	Enzo		2x5min.	5min.
M30	TecoMedical	Ms. mono.	pH 9.0; 20 min. WB 96°C	/4500	30min. RT	ZytoChem Plus AP	Zytomed	Kit	2x5min.	5min.
pSTAT3	Cell Signaling	Rb. mono.	pH 9.0; 20 min. WB 96°C	/50	60min. RT	ZytoChem Plus AP	Zytomed	Kit	2x5min.	5min.
pNRF2	Novus	Rb. mono.	pH 9.0; 40 min. WB 96°C	/25	60min. RT	Polyview Plus AP Rb	Enzo		2x5min.	5min.
Syntaxin	Sigma	Rb. poly	pH 9.0; 20 min. WB 96°C	/200	60min. RT	ZytoChem Plus AP	Zytomed	Kit	2x5min.	5min.
SGLT2	Novus	Rb. poly	pH 9.0; 20 min. WB 96°C	/50	30min. RT	ZytoChem Plus AP	Zytomed	Kit	2x5min.	5min.
SOCS3	Abcam	Rb. mono	pH 9.0; 20 min. WB 96°C	/100	30min. RT	ZytoChem Plus AP	Zytomed	Kit	2x5min.	5min.

Figure Legends

Supplementary Table S1: Antibodies and staining protocols are shown in detail. From each FFPE tissue 1-to-2 µm-thick sections
were cut, dewaxed and pre-treated for further processing in an automated staining device (Dako Autostainer, Dako Glostrup, Denmark).

Abbreviations: Rb: rabbit; Ms: mouse; Gt: goat; poly: polyclonal; DAB: 3,3′-diaminobenzidine ; pSTAT3: Phospho STAT3 ;
pNRF2: Phospho NRF2; mono: monoclonal; WB: water bath; HIER: heat-induced epitope retrieval.

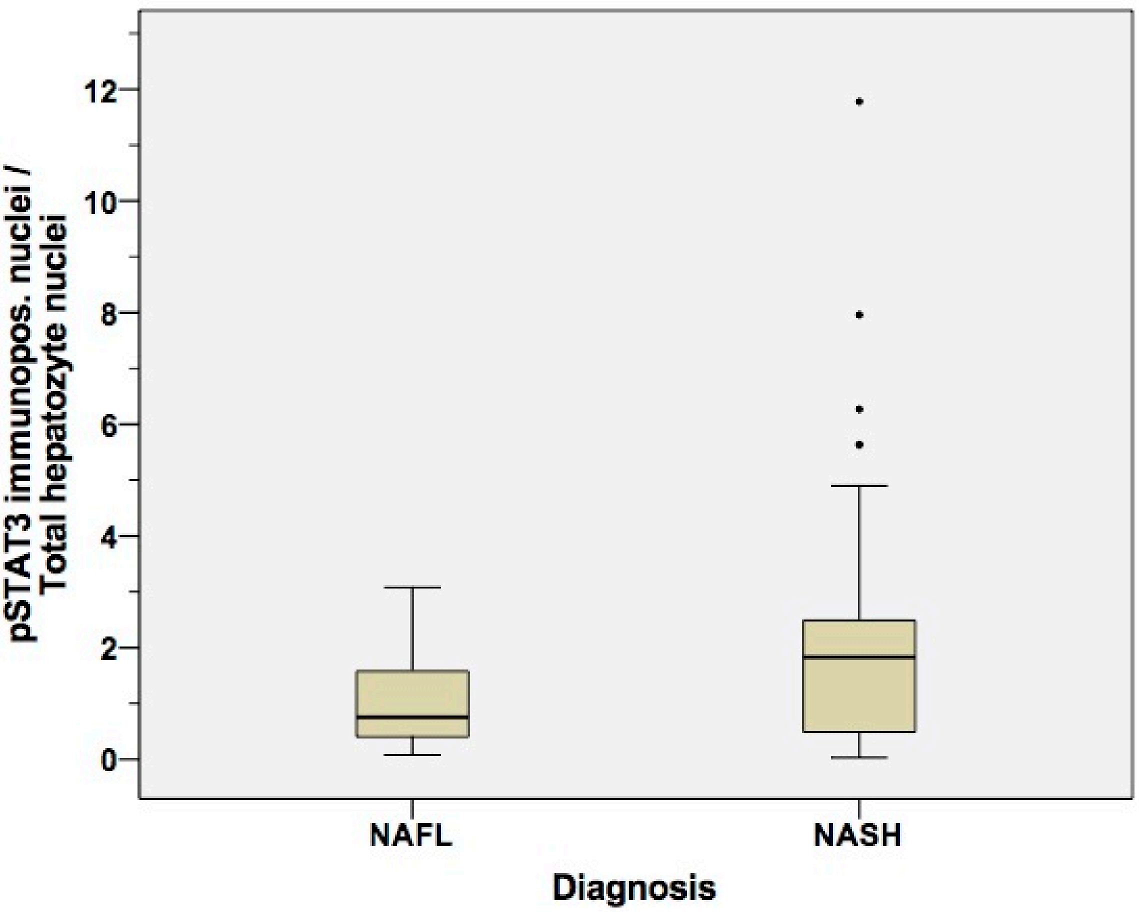

Supplementary Figure S1. Nuclear pSTAT3 immunostaining shows a trend toward higher values in NASH than in NAFL; p = 0.059.
